# Onset PrevenTIon of urinary retention in Orthopaedic Nursing and rehabilitation, OPTION—a study protocol for a randomised trial by a multi-professional facilitator team and their first-line managers’ implementation strategy

**DOI:** 10.1186/s13012-021-01135-x

**Published:** 2021-06-26

**Authors:** Ann Catrine Eldh, Eva Joelsson-Alm, Per Wretenberg, Maria Hälleberg-Nyman

**Affiliations:** 1 Department of Health, Medicine and Caring Sciences, Linköping University, SE-581 83 Linköping, Sweden; 2 Department of Public Health and Caring Sciences, Uppsala University, Box 564, SE-751 22 Uppsala, Sweden; 3 Department of Clinical Science and Education, Södersjukhuset, Karolinska Institutet, Södersjukhuset, SE-118 83 Stockholm, Sweden; 4 Faculty of Health and Medicine, Department of Orthopedics, Örebro University, SE-701 82 Örebro, Sweden; 5 Faculty of Medicine and Health, School of Health Sciences, Örebro University, SE-701 82 Örebro, Sweden

**Keywords:** Evidence-based practice, Guideline, Facilitation, Implementation, Leadership, Nursing, Orthopaedic care, Rehabilitation, Urinary retention

## Abstract

**Background:**

The Onset PrevenTIon of urinary retention in Orthopaedic Nursing and rehabilitation, OPTION, project aims to progress knowledge translation vis-à-vis evidence-based bladder monitoring in orthopaedic care, to decrease the risk of urinary retention, and voiding complications.

Urinary retention is common whilst in hospital for hip surgery. If not properly identified and managed, there is a high risk of complications, some lifelong and life threatening. Although evidence-based guidelines are available, the implementation is lagging.

**Methods:**

Twenty orthopaedic sites are cluster randomised into intervention and control sites, respectively. The intervention sites assemble local facilitator teams among nursing and rehabilitation staff, including first-line managers. The teams receive a 12-month support programme, including face-to-face events and on-demand components to map and bridge barriers to guideline implementation, addressing leadership behaviours and de-implementation of unproductive routines. All sites have access to the guidelines via a public healthcare resource, but the control sites have no implementation support.

Baseline data collection includes structured assessments of urinary retention procedures via patient records, comprising incidence and severity of voiding issues and complications, plus interviews with managers and staff, and surveys to all hip surgery patients with interviews across all sites. Further assessments of context include the Alberta Context Tool used with staff, the 4Ps tool for preference-based patient participation used with patients, and data on economic aspects of urinary bladder care.

During the implementation intervention, all events are recorded, and the facilitators keep diaries.

Post intervention, the equivalent data collections will be repeated twice, and further data will include experiences of the intervention and guideline implementation.

Data will be analysed with statistical analyses, including comparisons before and after, and between intervention and control sites. The qualitative data are subjected to content analysis, and mixed methods are applied to inform both clinical outcomes and the process evaluation, corresponding to a hybrid design addressing effectiveness, experiences, and outcomes.

**Discussion:**

The OPTION trial has a potential to account for barriers and enablers for guideline implementation in the orthopaedic context in general and hip surgery care in particular. Further, it may progress the understanding of implementation leadership by dyads of facilitators and first-line managers.

**Trial registration:**

The study was registered as NCT04700969 with the U.S. National Institutes of Health Clinical Trials Registry on 8 January 2021, that is, prior to the baseline data collection.

Contributions to the literature

- Despite guidelines for preventing and managing risks for urine voiding issues associated with hip surgery, the adoption into practice is lagging.
- This protocol delineates a trial with local facilitator teams across 10 orthopaedic sites supported to facilitate implementation of urinary retention guidelines during a year, whilst 10 control sites have regular access to the guidelines only.
- The study will investigate the effects of tailored implementation strategies on staff guideline adherence, the incidence of voiding complications before and after the intervention and between intervention and control sites, and orthopaedic patients, staff, and managers’ experiences of evidence-based voiding practice and guideline implementation.

## Background

Hip surgery is a common procedure in healthcare, performed for individuals with a hip fracture or as elective hip arthroplasty. In Sweden, with a population of just over 10 million people, about 18,000 individuals undergo hip fracture surgery each year [1, 2], and another 18,000 people undergo elective hip replacement surgery [3]. Hip surgery exposes the individual to an increased risk for urinary retention from bed rest, lack of privacy when toileting, pain, medication by means of opioids, anaesthesia and intravenous treatment with fluids—that is, factors associated with being an orthopaedic patient as well as known to cause urinary retention [4, 5]. In fact, the incidence of urinary retention in orthopaedic patients is notable: 7–44% of this group of patients are estimated to develop urinary retention prior to, during or post hip surgery [6–10], with studies reporting up to 84% of hip replacement patients having urinary retention [9, 11]. Although adherence to evidence-based practice guidelines would likely facilitate a decrease in urinary retention risks, there are indications that nursing and rehabilitation staff have inadequate knowledge and lack awareness of risks associated with urinary retention and the need for safer voiding care [12, 13].

Two decades ago, many healthcare professionals and decision-makers were roused by reports on the lack of evidence-based practice in healthcare [14, 15]. Despite progress, there are still gaps between what is known as best care and the healthcare provided for patients in certain areas [16]: in cases when urinary retention is not assessed and managed properly, the patient is exposed to a urinary incontinence, a permanent need for indwelling urinary catheterisation, and/or urinary tract infections or sepsis [17]. Though cost-effective and simple measures are available [18, 19], a general recognition of and adherence to these guidelines is lacking [20]. So far, there have been limited activities to address the implementation of these guidelines; such ventures imply local facilitator team and leadership engagement [21].

### Previous studies

The OPTION study originated from a European multi-centre study [13, 22–24] indicating that a earlier hip surgery episode had triggered urinary incontinence among frail older people residing in nursing homes. A subsequent pilot study in two Swedish orthopaedic units indicated that the knowledge and recognition of urinary bladder issues associated with hip surgery had not transferred to the nursing and rehabilitation staff [12]. Whilst a 3-month intervention to facilitate guideline implementation was appreciated and rendered an enhanced awareness of risk factors associated with hip surgery, the reach beyond the local facilitator teams was limited. Rather, the local teams (including a registered nurse, a physiotherapist or an occupational therapist in each site, plus a licensed practical nurse if so desired) engaged in the implementation programme, but for them to facilitate the uptake of evidence on urinary incontinence prevention in their sites, extended support beyond the 3 months with managers’ backing was required [25].

Parallel and later pilot studies in Sweden and Canada also showed that single professional facilitators or teams render some success in guideline implementation, whilst managers enacting as facilitators suffice in other instances—yet neither can singlehandedly address all essential elements [26–28]. Supposedly, a mutual effort is likely needed. Further, these studies emphasise the need to tailor facilitation activities to address the local context, as suggested by concurrent research [29]. Hence, the OPTION project enlists local facilitator teams across the intervention sites, including professionals and first-line managers, to embark on an extended and tailored implementation programme with support of external facilitators on the research team.

## Methods

### Aims

**Fig. 1:**
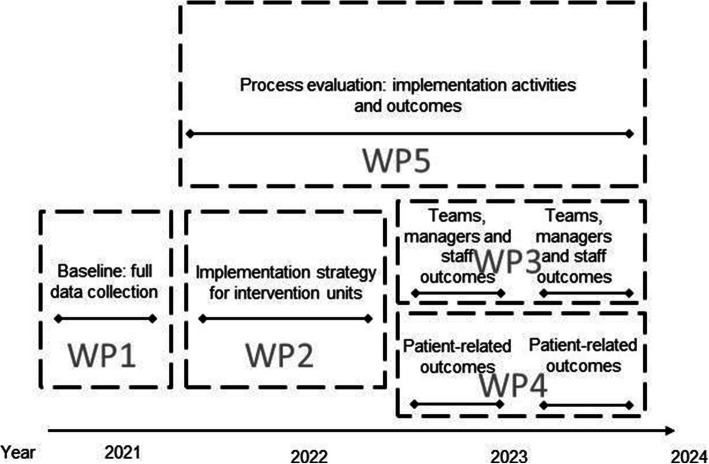
The OPTION project work packages: overall focus, and timeline (WP, work package)

The OPTION trial will evaluate the effects and outcomes of an implementation strategy for evidence-based practice by means of clinical practice guidelines for postoperative bladder distension and urinary retention. OPTION comprises five work packages, as illustrated in Fig. 1. The study hypothesis is that the adoption of guidelines in daily practice and de-implementation of procedures not compatible with evidence-based urinary retention guidelines will increase with local assigned teams including staff and managers guided to facilitate such implementation (compared with control sites receiving no support). Further, the hypothesis includes that the implementation of the national urinary retention guidelines will render increased prevention and detection of urinary retention in patients having hip surgery, leading to a decreased risk for bladder distension and subsequent lower urinary tract symptoms among patients in the study sites randomised to the support programme.

The specific aims of each work package of the project are as follows:

1. To investigate the incidence of urinary retention, bladder distension and complications among hip surgery patients; the procedures to prevent, detect, and manager urinary retention performed by staff; the hip surgery patients’ experience of voiding issues during the orthopaedic care episode, including the conditions for participation in such issues; staff and managers’ experience of guideline implementation in general and in urinary retention in particular; and staff experience of contextual factors known to influence knowledge implementation (WP1).
2. To provide an implementation support programme for the local facilitator teams, including managers, for 12 months to the sites randomised to the intervention (WP2).
3. To explore the outcomes in terms of attitudes and behaviours in terms of urinary retention and voiding care among staff and managers post intervention, including the procedures to prevent, detect, and manager urinary retention performed by staff, comparing the intervention sites with the control sites (WP3).
4. To explore the outcomes in terms of the incidence of urinary retention, bladder distension and complications among hip surgery patients during and after the hospital care episode, and the hip surgery patients’ experience of voiding issues during and after the orthopaedic care episode, including the conditions for participation in such issues (WP4).
5. To evaluate the activities of the support programme in relation to the contextual factors of the orthopaedic sites, and the activities and actors performed across intervention and control sites, respectively, identifying what influenced the process of facilitation implementation and de-implementation of evidence-based urinary retention care. This includes considering contextual factors, such as barriers and enablers for knowledge translation, and the cost associated with adhering to or diverging from guidelines (WP5).

### Overall design

**Fig. 2:**
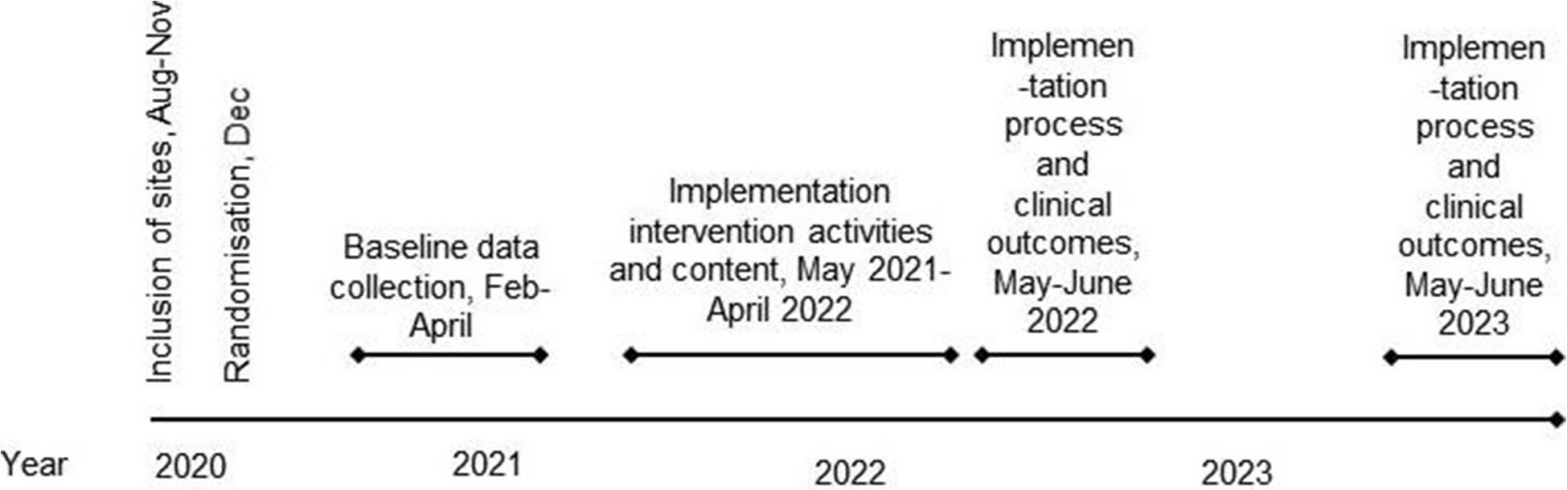
The overall points of time for inclusion, randomisation and data collections across OPTION

We propose a cluster-randomised trial with hybrid type III design, incorporating a twofold emphasis on the effectiveness of the implementation strategy (WP3 and WP4) and the outcome of the clinical intervention (WP4 and WP5) [30]; the scheme for crucial events is as in Fig. 2.

### Setting and inclusion criteria

The research team called upon orthopaedic units in university, regional, and local hospitals across Sweden. To avoid excess travel during data collections and enable participation in the intervention, the hospitals furthest north and south were contacted last. All 55 potential sites are equivalent in funding, either run by the public sector or company-driven hospitals with regional procurements. Further, they were considered equal, regardless of providing hip fracture surgery and elective hip replacements or elective hip replacement surgery only. A sample of 20 orthopaedic units was deemed necessary to provide sufficient statistical strength to detect an impending increase in adherence to the guidelines in the intervention group.

The sites were contacted by the research team with information and were engaged as a result of each head of department consenting to the OPTION study. Reasons for declining to participate concerned primarily staffing issues or heavy workload due to the pandemic (in some cases requiring orthopaedic units to transform into COVID-19 units).

### Power and sample

The study power calculation is based on opportunities to detect an impending increase in adherence to national bladder monitoring guidelines: assuming an absolute increased adherence to the guidelines of 15% (50% in the control group and 65% in the intervention), the required sample size is 227 patients in each arm, with 90% power and two-sided significance level of 0.05 (based on an intracluster correlation coefficient of 0.03). With 10 clusters in each arm and an average cluster size of 70 patients, a total of 1380 patients are needed. Considering a dropout rate of 10%, a total sample size of 1500 is required, providing an average cluster size of 75 patients. With a variance between around 5% and 80% incidence of urinary retention among hip surgery patients, it is difficult to calculate power based on this factor. Yet, considering that it may be 35–40%, the cluster size suggested above will also allow detecting a 15% absolute difference in incidence of urinary retention between the intervention group and the control group post intervention.

Once the units had agreed and the ethics board had approved the trial, the randomisation was performed. To prevent bias in terms of complexity of the patients’ medical status, and influence of clinical training programmes for students in nursing science and physio- and occupational therapy, university hospitals were equally distributed as intervention and control units. Further, two units located in Stockholm, the capital of Sweden, were randomised to either intervention or control. Thus, the randomisation was completed in four steps: university units, units in the Stockholm catchment area, and regional orthopaedic and local orthopaedic units, respectively, drawn from sealed envelopes by an impartial researcher engaged for this procedure whilst monitored over video link by the research team.

### Participants

#### Patients

Patients undergoing hip surgery in any of the orthopaedic units (whether control or intervention groups) during the project’s three data collection points will be informed about and asked to participate in the study at baseline and follow-ups I and II. Although the risk for urinary retention increases with age, and most hip surgery patients are becoming of advanced age, we will not exclude younger patients and thus discriminate due to age. Rather, inclusion criteria besides being admitted for hip surgery encompass the ability to communicate in Swedish without an interpreter, whilst a diagnosed cognitive impairment is an exclusion criterion.

#### Staff

All nursing and rehabilitation staff employed in the intervention and control units, respectively, are informed about the study; thus, registered nurses, licensed practical nurses, physiotherapists, and occupational therapists are invited to take part in the three data collection points, with the exclusion of those on long-term absence (e.g. parental or sick leave).

#### Managers

All managers of nursing and rehabilitation staff are likewise informed about the study and invited to take part in the data collection.

#### Internal facilitators

The staff assigned as internal facilitators (IFs) are recruited by the intervention units themselves, according to a document providing a background regarding facilitation, the IF’s qualifications in general and in OPTION, and suggesting selecting IFs based on:

- An interest in urinary retention and/or an interest in and ability to support others in evidence-based practice,
- A formal or informal leader recognised to enable improvement efforts,
- Being inclined to problem-solving and reflection, and,
- Being willing and capable of collaborating and learning.

The IFs are expected to take on their role for the 12-month intervention period and thus to hold a permanent employment position.

#### First-line managers

The first-line managers of the nursing and rehabilitation staff, respectively, will be expected to join the IF teams for the 12-month intervention period in the intervention units.

### The implementation object

The Handbook for Healthcare is a quality-assured resource for all healthcare workers and organisations across Sweden. It is provided by the Swedish Association of Local Authorities and Regions, that is, the public sector body for all health and medical services and social services. It comprises 122 chapters on fundamentals of care, including basic and advanced procedures. Whilst formerly a printed book, it has been online since 2002: vardhandboken.se. One of the chapters is ‘Bladder monitoring in hospital care’, including eight sections: an overview, symptoms and diagnosis of bladder extension, ultrasound with a portable bladder scan, assessment and interventions when a risk of urinary retention, schedule for voiding monitoring, bladder monitoring during surgery procedures, references and regulations, and a knowledge self-test on the chapter and its sections. The current chapter was revised in 2019 by two experts, both senior researchers and clinical experts, and reviewed by two independent experts in the area, comprising nursing and medicine.

### The implementation strategy

The OPTION intervention builds on the renowned Integrated Promoting Action on Research Implementation in Health Services (i-PARiHS) framework [31, 32] and the Ottawa Model of Implementation Leadership, O-MILe [33–35]:

The OPTION study trials a multi-professional, multi-level strategy: the orthopaedic units in the intervention group select their teams as above and are expected to serve as facilitators as individuals and as a group, enabling others to adapt the guidelines and addressing attitudes and routines that can pose barriers to such adaptation, including de-implementation of outdated or redundant praxis [29]. The 10 intervention units will receive the support programme for teams, whilst the 10 control group units will receive no support. Nevertheless, both the intervention units and the control units have and have had unlimited access to guidelines as described above.

**Fig. 3:**
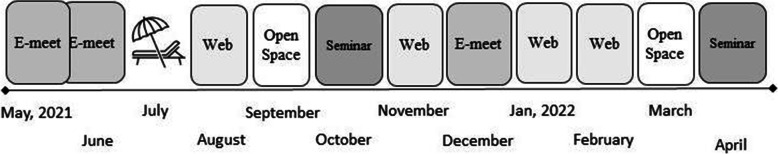
Overview of intervention timeline and activities

An overall plan for the events and timing is provided in Fig. 3.

- E-meet and seminar. Half-day or full-day seminars for all IF teams (including first-line managers) via video link or face-to-face venues. Scaffolding the local team and overall team building, some sessions will be for both IFs and managers, and some separate, addressing more specifically the IFs in terms of the clinical issues re urinary retention and knowledge translation [36, 37] and the managers in implementation leadership [26–28]. The purpose is for the IFs and managers to learn and share, including progress and experiences.
- Web. Providing the teams with tools and sources via the unique web page which they can access 24/7, with opportunities to post queries, this will provide on-demand support during the intervention and also build a resource bank, including FAQs, on guideline implementation and de-implementation of low-value care related to urinary retention matters.
- Open space. Opportunities to meet with the researchers (i.e. external facilitators) to consider and settle local issues and processes for implementation of evidence-based urinary retention care.

### Data collection

The primary outcome measure for OPTION is:

- Adherence to urinary retention guidelines in the care of patients undergoing hip surgery.

Additional outcomes are:

A. Incidence of and severity of urinary retention, bladder distension, and voiding complications among hip surgery patients (WP1, WP4 and WP5).
B. Activities and experiences of urinary retention, bladder distension, and voiding complications care among nursing and rehabilitation staff in orthopaedic care (WP1, WP3, and WP5).
C. The IFs’ and managers’ experiences of the facilitation programme (WP2 and WP5).
D. The patients’ experience of bladder care and voiding self-care management during the hospital stay and post discharge, including conditions for participating in these health-related issues (WP1, WP4, and WP5).
E. Use and costs of urinary retention devices and labour related to bladder monitoring (WP5).

Hence, data will be collected as follows:

A. Quantitative data regarding urinary retention will be collected from patient records, including bladder monitoring, voiding and healthcare interventions, by means of a structured template [12, 22] at baseline, post completion of the study intervention, and an additional, 1-year follow-up.
B. Structured patient record assessment in triangulation with qualitative interviews with a random sample of nursing and rehabilitation staff and managers across all sites [13, 23, 25, 38]. In addition, baseline data collection includes mapping the context, with the Alberta Context Tool (ACT) [39, 40] distributed among all nursing and rehabilitation staff.
C. Qualitative interviews post intervention (follow-ups I and II), in addition to voice recordings of all events during the intervention (support programme) [13, 23, 41]. Further, IF teams and managers will be asked to keep notes, by means of an individual, semi-structured reflective diary [42].
D. Structured patient record assessment in triangulation with a post-discharge structured survey to all hip surgery patients for voiding difficulties after the hospital episode, self-care management and conditions for participating in accordance with one’s preferences—the latter using the 4Ps tool (Patient Preferences for Patient Participation) [43–45]. Further, data will originate from qualitative interviews with a sample of two patients per data collection point and site, representing the age range and gender distribution among those having had hip surgery.
E. The structured patient record assessments at baseline, follow-up I and follow-up II will inform the health economics evaluation.

### Data analysis

Data from patients’ records, the Alberta Context Tool questionnaire, the patient survey on voiding difficulties, and patient participation will be analysed with statistical analyses [46]. Between-group differences will be analysed using Student’s independent-sample t test for continuous variables, the Mann–Whitney U test for non-normally distributed continuous variables, and a χ2 test for nominal variables.

Qualitative content analysis will be applied for all interviews [47, 48], subsequently transcribed verbatim to text files by a professional service.

All data will be considered in the final mixed methods [49], embedding the process evaluation [50].

## Discussion

The OPTION project addresses elements of necessity to progress implementation science and clinical practice: team strategies for guideline implementation, including the managers’ role and function in relation to IFs, and barriers and enablers for safe bladder management in the orthopaedic care context. As such, it requires vigilant data collection and analysis [51], considering the theoretical and practical aspects of epistemology and methodology. Whilst we draw on previous studies, we expect the OPTION study to advance research issues by considering the effects of the implementation strategy for both patients and professionals [42].

For patients, increased adherence to guidelines for voiding care in the orthopaedic context can decrease the risk of adverse events and complications associated with hip surgery [52], particularly the often preventable risk of urinary retention [53]. Further, advanced interprofessional teamwork to address urinary retention is likely to incorporate the patient perspective, enhancing opportunities for individuals to participate in issues regarding their health and healthcare [43]. Whilst clinical measures are needed to determine the impact of the OPTION implementation strategy, the evaluation will augment the understanding of patient-related outcome measures in implementation science. In addition, healthcare professionals will have an option to progress from knowing what to do—learning about the readily available national guidelines—to providing orthopaedic nursing and rehabilitation care according to evidence-based standards. Any such change signifies altered clinical routines and modified attitudes and behaviours [25] addressed by the implementation strategy. If facilitating adoption of guidelines, the experiences of OPTION can be applied for knowledge translation in orthopaedic care and other surgical settings, across and beyond the participating hospitals and Swedish healthcare [54]. In the OPTION trial, details needed for a transparent trajectory in terms of the implementation process and outcomes are considered, particularly how the relations between the evidence, the facilitation, and the context can explain the outcomes [55]. For now, the lack of evidence-based procedures and the use of dated knowledge in health care cause substantial suffering for individual patients, at far too high a proportion of healthcare budgets.

## Data Availability

The data collection tools developed for the OPTION trial are available upon reasonable request from author MHN, except for the 4Ps tool, which is available from author ACE. The Alberta Context Tool is used by courtesy of Professor C. Estabrooks, University of Alberta, Canada.
